# Supplementary material for: Are carbon emissions trading and green financial instruments synergistic? -Comprehensive quantitative research based on content analysis
Source: PLoS One. 2024 Mar 7;19(3):e0298601. doi: 10.1371/journal.pone.0298601 (PMC10919601; doi:10.1371/journal.pone.0298601)
Supplement: S1 Data — (ZIP) [file pone.0298601.s001.zip › Data/地级市Z_F工作报告.zip!/╒π╜¡/║╝╓▌╩╨/└┤╫╘╣1⁄2╓┌║┼í╛┬φ┐╦╩2╛▌═°í┐_2016.docx]

各位代表：

　　现在，我代表市人民政府向大会报告工作，请予审议，并请市政协委员和其他列席人员提出意见。

　　一、2015年工作和“十二五”发展回顾

　　2015年是不平凡的一年。市政府认真贯彻落实党中央国务院、省委省政府和市委各项决策部署，主动适应发展新常态，突出改革创新、转型升级、环境治理、民生改善、社会和谐等工作重点，励精图治、奋发进取，全面完成了市十二届人大五次会议确定的各项目标任务。尤为可喜的是，在党中央国务院关心支持和省市共同努力下，杭州成为2016年G20峰会举办城市，并成功获得2022年亚运会主办权、获批设立中国（杭州）跨境电子商务综合试验区和建设杭州国家自主创新示范区，为推进我市高起点上新发展注入了强大动力。

　　（一）转型升级加快推进，发展质效持续提升。坚持稳增长与调结构相结合，经济运行高开稳走向好，全市生产总值10053.58亿元，成为全国第十个总量超万亿元城市，比上年增长10.2%，增幅居全省第一、副省级以上城市第二；财政总收入2238.75亿元，其中一般公共预算收入1233.88亿元，分别增长11%和9.8%。投资结构进一步优化、重大项目有力推进，全市完成固定资产投资5556.32亿元，增长12.2%。成功举办西博会、动漫节、文博会、电商博览会、国际快递业大会，全市社会消费品零售总额4697.23亿元，增长11.8%，网络零售额增长42.6%；出口总额（不含省属企业）2760.72亿元，增长5.1%，其中跨境电商出口141.13亿元。实际利用外资71.13亿美元，增长12.3%；国内招商到位资金1249.27亿元，增长18.5%，其中浙商回归到位资金660.58亿元，增长27%，成功引进和签约一批重大产业项目。深入实施“一号工程”，信息经济强势发展，高新技术产业、战略性新兴产业、先进装备制造业增加值增幅明显高于规上工业，电子商务、文化创意、旅游休闲、金融服务等优势产业快速发展。新增境内外上市企业9家、新三板挂牌企业130家。新建省级现代农业园区18个、各级粮食生产功能区262个，现代都市农业发展取得新进展。

　　（二）创新驱动持续强化，发展动力加快转换。坚持把创新驱动作为发展主引擎，全市一般公共预算科技支出增长33.9%，入选中国十大创新生态城市，成为首批国家小微企业创业创新基地示范城市，大众创业万众创新呈现良好态势。临江国家高新区获批，杭州高新区（滨江）、杭州经济开发区、城西科创产业集聚区、大江东产业集聚区等平台创新集聚功能增强，新增国家重点扶持高新技术企业311家，全市高新技术产业增加值达到1212.60亿元，贝达药业小分子靶向抗癌药项目获国家科技进步一等奖。纳入国家科技孵化器体系的众创空间达到14家，约占全国总量的十分之一；省级特色小镇达到9个、市级特色小镇32个，成为创业创新重要载体和经济新增长点。落实“人才新政27条”，发放人才创新创业资助资金1.4亿元，办理人才落户10730名。新增大学生创业企业 1485家。科技、产业、金融深度融合发展，新增备案私募金融机构838家，新成立蒲公英等引导基金10家。成功举办2015杭州云栖大会，全国云计算中心地位初步确立。

　　（三）重点改革深入实施，发展活力明显增强。跨境电商综试区建设取得重大成效，一批管理制度和试点经验得到国务院高度肯定，并向全国复制推广。深化“四张清单一张网”改革，浙江政务服务网（杭州市）运行的行政许可事项达554项。开展行政规范性文件专项清理，废止和宣布失效文件543件。工业企业“零土地”技术改造项目不再审批。实现“五证合一、一照一码”企业注册登记制度全覆盖，全市新登记企业62998家、新增注册资本4529.45亿元，分别增长17.6%和56.9%。新设“七大产业”小微企业20922家，新增“个转企”3954家、“小升规”1852家、省股权交易中心“挂牌”企业450家。投融资体制改革取得新进展，筹建设立规模逾400亿元的城市发展基金。全市消化利用批而未供土地4.52万亩、低效用地再开发3.86万亩。大江东管理新体制实体化运作，余杭经济开发区与钱江经济开发区整合提升，钱塘智慧城启动建设，杭汽轮等市属国企混合所有制改革加快。出台实施出租汽车行业管理体制和运营机制改革方案。开展公共租赁住房货币补贴试点，出台拆迁安置住房保障货币化政策。居民用水和天然气价费改革平稳推进。成为首批全国社会信用体系建设示范城市。

　　（四）城乡统筹发展加快，功能品质持续提升。杭州城市总体规划（修订）获得国务院批复。G20杭州峰会筹备工作顺利开展，场馆设施改造提升和环境重点整治项目有力推进。杭新景高速公路建德段等市域交通项目竣工，东湖快速路、文一路地下通道等市区交通项目快速推进，秋石三期四期、环城北路地下通道、吉鸿快速路等治堵重点工程建成开通。地铁四号线首通段、一号线下沙延伸段建成运营，轨道交通初步成网。打通“断头路”12条，新建停车泊位50156个。推行公交优先优惠新措施，主城区公共交通分担率有新提升。道路分类保洁项目荣获中国人居环境范例奖。全国社区治理和服务创新实验区通过考核验收。深入实施城乡区域统筹“六大西进”行动，落实区县（市）协作资金3.78亿元、项目92个，落实联乡结村帮扶资金1.68亿元。农村“三权一房”确权登记颁证和农村供销、生产、信用合作“三位一体”改革深入推进，村级股份制改革进一步深化。加快建设美丽县城、小城市和中心镇，推进193个中心村和62个美丽乡村、精品村建设，开展12个杭派民居建设试点，培育民宿示范村(点)57个，乡村旅游（民宿）游客突破3000万人次。

　　（五）生态保护持续加强，环境整治成效明显。落实环境目标责任，加强生态环境建设，国家生态文明先行示范区建设扎实推进。市本级通过国家生态市技术评估，萧山、富阳通过国家生态区考核验收，桐庐、淳安入选首批国家生态保护与建设示范区，临安市成为“国家园林城市”，杭州经济开发区被评为国家生态工业示范区。深化“五水共治”，千岛湖配供水工程有序推进，闲林水库下闸蓄水。实现1845条乡镇级以上河道“河长制”全覆盖，完成 84条137公里黑臭河整治、16座污水处理厂提标改造，新增污水管网239公里；农村生活污水治理保持全省领先。市控以上断面水质达标率85.1%，交接断面水质获省考核优秀。节水技术改造持续推进，全市万元生产总值用水量预计下降6%。完成防洪排涝项目221个，三堡排涝工程建成并在汛期发挥重要作用。加快“五气共治”，杭钢集团转型升级取得实质性突破，半山和萧山电厂燃煤发电机组关停，主城区“无燃煤区”基本建成，淘汰黄标车81079辆，率先成为无钢铁生产企业、无燃煤火电机组、无黄标车“三无”城市。投入使用新能源汽车11053辆，累计达22131辆，总量居全国城市第三。全市建筑工地实施全方位扬尘管理。全年环境空气优良天数242天，比上年增加14天，PM2.5浓度同比下降12.3%。推进“五废共治”，处置各类垃圾和废物552.62万吨。完成“三改”2168.85万平方米、拆违2057.89万平方米，完成“两路两侧”“四边三化”专项问题自查和整治。关停转迁落后产能企业426家，单位生产总值能耗预计下降3%。节能减排财政政策综合示范工作获得全国优秀。严厉打击污染环境违法犯罪行为，查办案件67件，判处罪犯156名。

　　（六）为民惠民不断深化，保障水平有效提高。坚持民生为本，十件实事全面完成。全市一般公共预算民生支出921.92亿元，占一般公共预算总支出的比重超过75%。城镇、农村居民人均可支配收入分别增长8.3%和9.2%，家庭人均收入低于4600元的贫困现象全面消除。新增城镇就业28.79万人，帮助失业人员再就业14.22万人，城镇登记失业率1.74%。棚户区改造和保障性安居工程开工47394套、竣工57530套，提供经济适用住房配售房源2637套，推出市本级公共租赁住房（廉租住房）配租房源10821套。新增机构养老床位5782张，改扩建居家养老服务照料中心774家。全市新增中小学、幼儿园73所，新增外籍人员子女学校3所，义务教育基本均衡县实现全覆盖。主城区中小学、幼儿园名校（名园）集团化覆盖面分别达到79.7%和71.2%，义务教育学校配套建设三年行动计划全面启动实施。浙江音乐学院建成使用。杨金龙获第43届世界技能大赛金牌。成为全国城市公立医院综合改革试点，医养护一体化全科医生签约人数达52万人；市中医院丁桥分院等4所市属重点新医院加快建设，新建和改造残疾人庇护中心、康复托养机构245家。出台实施特殊药品大病保险政策。群众文化建设继续加强，新建农村文化礼堂107个。“最美现象”不断涌现，市民素质和文明程度有新提升。开展严厉打击危害食品药品安全、通信和网络诈骗、“黄赌毒恶”等违法犯罪专项行动，加强社会治安综合治理，全市刑事案件继续实现命案全破。加强重点行业领域事故隐患排查治理和打违除患行动，各类事故起数、死亡人数连续12年下降，平安杭州建设取得积极成效。

　　同时，我们积极开展友好城市合作交流，扎实做好山海协作、对口支援帮扶等工作。外事、港澳台事务、侨务、民族宗教、双拥优抚、国防动员、人防、地方志、气象、残疾人、慈善、红十字、老龄、妇女儿童、关心下一代等工作都取得了新发展。

　　各位代表，2015年各项工作的扎实成效,为圆满收官“十二五”作出了积极贡献。回顾过去五年，我们战胜了诸多困难和风险，经受住了严峻挑战和考验，通过全市上下的不懈努力和共同奋斗，杭城大地发生了新变化、增添了新活力，经济社会发展迈上了新台阶。

　　五年来，我们坚持科学发展、富民强市，全市生产总值从2010年的5965.71亿元跃升至2015年的10053.58亿元，年均增长9.1%；常住人口人均生产总值18025美元，达到高收入经济体水平；一般公共预算收入年均增长12.9%；“一基地四中心”城市功能建设取得重大进展，全市综合实力和竞争能力大幅提升。

　　五年来，我们坚持创新创业、转型升级，全社会研发经费支出从166.9亿元增加到301.6亿元；累计引进海外留学人员2.3万人；累计自主申报入选国家“千人计划”94名，居副省级城市第一；专利授权累计19.12万项，其中发明专利授权2.88万项，连续11年位居省会城市第一；高新技术企业从2285家增加到4044家，高新技术产业增加值占规上工业比重从27.1%提升到41.8%，“十大产业”占全市生产总值比重达到52%；三次产业结构从3.5∶47.3∶49.2调整为2.9∶38.9∶58.2，经济发展新格局基本形成。

　　五年来，我们坚持深化改革、扩大开放，推进杭州都市经济圈转型升级综合改革、淳安生态功能区建设等国家和省级改革试点，实施大江东体制调整、萧山余杭与主城区一体化发展、富阳撤市设区、要素市场化配置等重大改革；市场主体从49.15万家增加到75.46万家，注册资本从8438亿元增加到23130亿元；国际友好城市达29个；累计到位外资284.06亿美元，30家世界五百强企业在杭投资项目58个；境内外上市、新三板挂牌企业分别达118家和155家；全国民营企业500强达55家，连续13年居全国城市第一，发展活力进一步显现。

　　五年来，我们坚持城乡统筹、市域协同发展，固定资产投资累计21595.67亿元，建成铁路杭州东站枢纽、钱江通道等一批重大交通项目；开启地铁时代，建成运营轨道交通82公里；“六大西进”、区县（市）协作和联乡结村成效明显，四县（市）地区生产总值、财政收入、固定资产投资、农村居民收入等指标增幅高于全市平均，城乡区域发展更趋均衡。

　　五年来，我们坚持生态优先、环境立市，“三江两岸”绿道全线贯通，深入开展“五水共治、五气共治、五废共治、三改一拆、四边三化、两路两侧”等环境治理专项行动，城乡环境面貌实现新改善，化学需氧量、氨氮、二氧化硫、氮氧化物排放量前四年累计分别下降16.3%、13.7%、15.4%、17.6%，提前一年完成任务；单位生产总值能耗五年累计下降22.5%左右，大气和水环境质量不断提升。

　　五年来，我们坚持以民为先、惠民安民，重点民生领域一般公共预算资金累计投入3389亿元，社会发展总指数居全国副省级城市首位。城镇居民人均可支配收入从30335元提高到48316元，年均增长9.8%；农村居民人均可支配收入从14778元提高到25719元，年均增长11.7%，城乡居民收入比从2.05：1缩小到1.88：1。基本养老、基本医疗保险参保人数分别达到668.65万人和870.71万人。西湖文化景观、大运河成功列入世界文化遗产名录，杭州成为“双世遗”城市。社会民生各项事业统筹推进，社会安定有序、人民安居乐业。

　　五年来，我们加强政府自身建设，认真开展党的群众路线教育实践活动和“三严三实”专题教育，深化作风建设，强化绩效管理，提升行政效能。全面推进依法行政，加快职能转变, 市级部门行政权力精减幅度达70.6%;提请市人大常委会审议地方性法规28件，累计制定和修改政府规章23件、办理人大建议和政协提案6258件，法治政府和服务型政府建设取得新进展。

　　各位代表，过去五年成绩的取得，是党中央、国务院亲切关怀的结果，是省委、省政府和市委坚强领导的结果，是全市干部群众团结奋斗的结果。在此，我代表市人民政府，向全市人民和外来建设者，向市人大代表和政协委员，向各民主党派、工商联、无党派人士、各人民团体和社会各界人士，向中央驻杭单位和省级各部门，向驻杭解放军和武警部队官兵，向关心支持杭州发展的港澳台同胞、海外华人华侨和国际友人，表示衷心的感谢和崇高的敬意！

　　我们清醒地认识到，杭州经济社会发展中还存在不少问题和矛盾，主要表现在：自主创新能力还不强，传统产业转型提升不够快；城市国际化程度还不高，城乡和区域发展还不够平衡；资源环境约束趋紧，环境质量提升压力较大；人口老龄化趋势加快，提高基本公共服务供给能力、改善民生的任务日趋加重；影响稳定的潜在风险和隐患较多，维护社会稳定和城市安全的压力加大。在政府自身建设方面，一些工作人员的改革意识、法治意识、服务意识、责任担当意识和行政能力还不强，一些领域的消极腐败现象还时有发生。对此，我们一定高度重视，采取有力措施，认真改进和解决。

　　二、“十三五”发展主要目标任务

　　“十三五”是充满机遇和挑战的五年，是杭州高水平全面建成小康社会的决胜阶段，也是加快经济转型、城市转型、社会转型、政府转型的攻坚阶段。根据中共杭州市委《关于制定杭州市国民经济和社会发展第十三个五年规划的建议》，市政府制定了《杭州市国民经济和社会发展第十三个五年规划纲要（草案）》，提请大会审议。经本次大会批准后，市政府将认真组织实施。

　　“十三五”发展指导思想是，高举中国特色社会主义伟大旗帜，以马克思列宁主义、毛泽东思想、邓小平理论、“三个代表”重要思想、科学发展观为指导，深入学习贯彻习近平总书记系列重要讲话精神，按照“四个全面”战略布局，坚持创新、协调、绿色、开放、共享的发展理念，持续深化“八八战略”实践，肩负“干在实处永无止境，走在前列要谋新篇”新使命，以改善民生为根本目的，以率先发展为第一要务，以改革创新为第一动力，以办好“两会”、建设“两区”为重要抓手，统筹推进经济、政治、文化、社会和生态文明建设，高水平全面建成小康社会，确保继续在全省发挥龙头领跑示范带动作用，确保继续走在全国重要城市前列，共建共享历史文化名城、创新活力之城、东方品质之城，努力建成美丽中国的样本，朝着建设世界名城目标大步迈进。

　　“十三五”发展主要目标是，力争提前实现地区生产总值、人均生产总值、城乡居民收入均比2010年“翻一番”，在全国全省率先高水平全面建成小康社会，为建设世界名城打下坚实基础。全市生产总值年均增长7.5%以上，到2020年，服务业增加值比重达到62%，居民人均可支配收入年均增长8%，社会保障覆盖面达到100%；研发经费支出占地区生产总值比重达到3.3%，高等教育毛入学率达到65%，使杭州综合实力更强、发展质效更高、城市功能更全、生态环境更优美、治理体系更完善、人民生活更美好。

　　为实现“十三五”目标，我们将坚持强活力、转动力，增优势、补短板，重协调、促均衡，重点抓好六个方面工作。

　　（一）提升城市国际化水平。推进城市国际化是杭州中长期发展的重大战略任务，必须加强城市硬环境、软实力建设，完善城市国际化支撑体系，提升国际竞争力，加快迈入世界名城行列。

　　打响城市国际化品牌。全力服务保障2016年G20峰会顺利召开，努力体现中国特色、中国风格、中国气派。全力承办好2017年全国学生运动会、2018年世界短池游泳锦标赛，有序推进2022年亚运会筹备工作，打造具有国际影响力的赛事之城。放大“后峰会、前亚运”效应，积极吸引国际组织地区总部和分支机构落户，提升城市国际知名度。

　　提升经济国际竞争力。更加积极融入全球创新网络，以电子商务、物联网、云计算和大数据、高端装备制造等领域为重点，培育一批国际知名创新型领军企业、打造产业高地，成为“互联网＋”国际引领城市。深入实施旅游国际化和旅游全域化战略，到2020年境外旅游者接待量达到435万人次以上，建设国际重要的旅游休闲中心。大力培育引进一批国际性会展项目和企业，提升展会国际化水平，打造国际会展之都。

　　增强文化国际影响力。实施“杭州城市记忆工程”，挖掘良渚文化、吴越文化、南宋文化等传统文化精髓，弘扬“精致和谐、大气开放”人文精神，彰显文化特质；加强历史文化名城保护，发挥西湖和大运河“双世遗”带动效应，推进跨湖桥、良渚、南宋皇城、钱塘江古海塘、西溪湿地等遗址的申遗和保护利用；加强非物质文化遗产保护传承和利用，扩大丝绸、茶叶、中医药等传统文化和产品的展示与输出，提升动漫影视等特色文化产业国际影响力，加强与国际友城合作，深化人文交流，成为东方文化国际交流重要城市。

　　增强环境国际吸引力。开展国际对标行动，实施国际化环境营造十大工程，加快重点领域国际化步伐，外籍人员子女学校达到8所，国际标准认证医疗中心和国际化医院达到8所以上。按照国际通行规则提高服务水平，完善城市标识系统。提升市民国际开放意识，增强城市文化多元性和包容性。建设国际性区域交通枢纽，杭州空港国际及地区通航点达到40个左右，打造亚太地区重要的国际门户。

　　（二）增强创新创业能力。把创新作为引领发展的核心动力，实施“创新创业新天堂”行动计划，打造中国创新创业之都。

　　加强平台创新。加快国家自主创新示范区“一区十片、多园多点”建设，高水平建设国家高新区和省级产业集聚区，整合提升各类科技园区（开发区），努力成为具有全球影响力的“互联网+”创新创业中心。规划建设城西科创大走廊、城东智造大走廊、钱塘江生态经济带、运河湖滨高端商务带、钱塘江金融港湾、杭州空港经济区，激发经济增长潜力和创新动力。培育建设特色小镇100个、市级众创空间100家、各类服务载体500家，努力成为大众创业万众创新的乐园。

　　加强科技创新。支持在杭高校、科研机构、企业研究院组建科技和产业联盟，提升科技成果转化能力，掌握一批关键核心技术和自主知识产权，建设创新型城市。到2020年每万人发明专利拥有量达33件，科技进步贡献率达到65%以上，高新技术产业增加值占规上工业增加值达到50%，培育科技型小微企业2万家以上，打造创新活力之城。

　　加强产业创新。深入实施“一号工程”，推进智慧产业化、产业智慧化，形成以信息经济为引领、高端服务业为主导、先进制造业为支撑、都市现代农业为基础的产业新体系。建设信息经济“六大中心”，力争信息产业集群企业主营业务收入突破万亿元；建设文化创意、旅游休闲、金融服务、高端装备制造、健康、时尚等六大千亿产业集群，力争六大产业增加值总量突破万亿元。推进工业化信息化深度融合，实施“互联网+”行动计划、“中国制造2025”杭州行动纲要，加快化纤纺织、轻工、汽配等传统优势产业转型升级，提升发展茶叶、丝绸、工艺美术等历史经典产业和特色产业。加快发展现代生态循环农业，率先基本实现农业现代化。

　　加强人才创新。实施新一轮全球引才“521”计划，充分发挥现有创新创业人才队伍的牵引作用，加强急需人才培养和引进，引进海外高层次人才2万名，引进和培育国内外顶尖人才20名、国家级领军人才500名，到2020年全市各类科技创业者超过30万人，高技能人才占技能劳动者比例达到30%，人才总量达到250万人，努力建设人才强市。

　　加强制度创新。健全“四张清单一张网”动态调整机制和监督落实机制，形成规范高效的事中事后监管体系。推进大部门制改革，推进跨部门跨领域综合执法体制改革，探索行政区划调整，推进县域综合改革。推进资源要素配置改革。深化投融资体制改革，加快国有资产证券化，基本完成竞争类国企混合所有制改革。建设信用杭州，打造全国社会信用体系建设示范标杆城市。推进军民融合深度发展。深化各类国家和省级改革试点，努力成为体制机制创新先行先试城市。

　　（三）提升城乡均衡协调水平。加大统筹协调力度，促进新型工业化、信息化、城镇化、农业现代化同步发展，努力建成经济繁荣、和谐宜居、生态良好、富有活力、特色鲜明的现代化城市。

　　优化区域空间布局。优化市域空间总体布局，编制市域空间规划，落实主体功能区制度，推进“多规合一”，促进生产空间集约高效、生活空间宜居适度、生态空间山清水秀。遵循城市发展规律，严格执行城市总体规划（修订），明确城市开发边界，加强空间管控，强化市区规划统一管理，促进城市紧凑发展。

　　深化城乡一体化。坚持“两疏散、三集中”，深化城市有机更新，推动主城区商品专业批发市场、学校和医院等功能向外疏解，加快跨江拥江发展，提升中心城市集聚功能和辐射带动能力。制定实施新一轮城乡区域统筹发展政策，加大产业转移和帮扶力度，发挥桐庐、淳安、建德、临安4县（市）自身优势，推动县域经济向大都市郊区经济转变，促进市域协同发展。提高乡村规划建设水平，推动杭派民居建设，建设一批高品质小城市、中心镇、特色镇和美丽乡村。

　　完善城乡基础设施体系。实施综合交通畅通工程，推进300余项交通重点项目建设，完成投资3500亿元，实现地铁网、城际铁路网、城市快速路网、高铁网、公路网、航道网、航空网互联互通。加强城市交通治理，形成“四纵五横”城市快速路网和建成250公里轨道交通骨架网络。合理利用地上地下空间，科学建设停车场（库）。加快电网改造升级，完善市域电网主网架结构。坚持公交优先，完善城市慢行系统和绿道网，建成“公交都市”和国家绿色交通发展城市。五年基本完成杭州主城区城中村改造。三年基本完成全市城镇危旧住宅房屋治理改造。推动实施老旧住宅小区电梯改造。推进智慧政务云为主体的基础平台应用，提升数字杭州地理系统建设服务水平，完善智慧城管、网上产品质量监管等协作平台，加快智慧城市建设。

　　（四）加快美丽杭州建设。坚定走“绿水青山就是金山银山”的科学发展之路，推进可持续发展，打造“两美”浙江示范区，努力成为美丽中国先行区。

　　加强生态保护和建设。坚守生态底线，划定生态红线，保护西部生态安全屏障，深化“四边三化”，构筑“两圈两廊多块六带”生态格局。全面深化城市、山区、平原和村庄绿化建设，巩固“江河湖海山田城”自然生态基础网架。

　　强化环境综合治理。深化“五水共治”，全面消除黑臭河和地表水劣V类水质，钱塘江杭州段、苕溪杭州段水质达到或优于Ⅲ类水。深化“五气共治”，PM2.5浓度实现明显下降，空气质量明显改善。实施“五废共治”，建成投运一批固废处理设施，提升治废能力。加快淘汰落后产能，完成主要污染物排放削减目标，打造环境监管最严格城市。

　　推进资源节约高效利用。发展生态经济和循环经济，推广绿色建筑和节能低碳技术。实行最严格的耕地保护制度，建立集约节约用地激励和约束机制，推动城区工业旧厂房和低端、零散产业用地改造整合，加快低丘缓坡土地开发利用，到2020年单位建设用地生产总值达到5.5亿元/平方公里，建设资源节约型城市。

　　健全生态文明制度体系。实施以单位生产总值能耗为基础的用能权有偿交易制度，推进新安江和千岛湖水环境补偿国家试点，建立生态文明绩效评价和责任追究制度，建立领导干部自然资源资产离任审计和生态环境损害责任终身追究制，建成国家生态文明先行示范区。

　　（五）营造开放合作新优势。以更高视野、更新姿态融入全球化，推进区域合作发展，努力成为“网上丝绸之路”枢纽都市。

　　发挥跨境电商综试区先发优势。深化全国首个跨境电商综试区建设，完善“六体系两平台”，发展“互联网+外贸+中国制造”模式，推进跨境电商自由化、便利化、规范化，打造全球最优的跨境电商生态圈，实现跨境贸易大发展。

　　加快对外贸易转型升级。推动海关特殊监管区域优化升级，争创杭州综合保税区。完善外贸综合服务平台，争取开展“市场采购”贸易方式，提高自有品牌、自主知识产权、自主营销产品和高新技术产品的出口比重，促进外贸提质增效。扩大服务贸易，发展服务外包，推进服务贸易与货物贸易协调发展，打造外贸综合竞争优势最强城市。

　　优化外资利用和对外投资。创新招商方式，引导外资投向，提升招商引资质量。实施新一轮“以民引外、以企引企”行动计划，支持民企以多种方式与外商合资合作，使杭州成为外商云集的投资热土。深入实施浙商回归工程，办好世界杭商大会，加强产业招商。鼓励支持企业“走出去”，培育一批具有全球竞争力和影响力的本土跨国公司。

　　深化区域合作。积极参与国家“一带一路”建设，主动融入长江经济带战略，加强对口支援各项工作，巩固提升“一基地四中心”功能，加快杭州都市区规划建设，提升杭州都市圈协同发展水平。

　　（六）共享幸福和谐品质生活。以人民为中心，深入推进生活品质共建共享，增加市民获得感，努力成为全面小康的品质之城。

　　促进社会就业和增收。完善就业创业培训教育服务支撑体系，提高全社会就业水平，新增城镇就业85万人。完善长效机制，多途径增加城乡居民收入，着力提高低收入群体的收入水平。

　　完善社会保障体系。实施全民参保计划，推进全市统一的社会保险管理信息系统建设，实现社会保险精细化管理。发展职业年金、企业年金、商业养老保险，探索建立长期护理保险制度。完善大病保险制度。完善城乡“四级救助”网络，扩大社会救助覆盖面。发展社会福利和慈善事业。

　　推进公共服务均等化。推进学前教育优质均衡发展、义务教育高水平均衡发展、普通高中教育多样化发展，加快教育现代化。加快现代职业教育发展，完善终身教育体系。加强公共卫生，深化医药卫生体制改革，推进医养护一体化，健全城乡基本医疗卫生制度。深入研究人口发展战略，发展健康服务和养老服务业，发展体育事业和体育产业，健全农村留守妇女儿童和老人关爱服务体系，支持发展残疾人事业，建设健康城市。加强精神文明建设，实施“满城书香”工程，加快公共文化事业发展，创建联合国教科文组织全球学习型城市。

　　建设平安和谐社会。深化“平安杭州”建设，完善立体化社会治安防控体系、基层社会治理网络体系，加强流动人口管理服务，鼓励支持社会组织参与社会治理。加强社区建设，规范社区管理。落实安全生产责任制，加强公共安全，保障食品药品安全。健全防灾减灾救灾机制，维护人民生命财产安全，打造最具安全感城市。

　　各位代表，宏伟蓝图已经绘就，只要我们坚定信心、埋头苦干，开拓创新、团结奋斗，“十三五”宏伟目标一定能转化为美丽现实！

　　三、2016年政府工作安排

　　2016年是“十三五”发展开局之年、G20杭州峰会举办之年、本届政府收官之年，全市改革发展稳定的任务十分繁重，机遇与挑战并存，必须保持清醒头脑，积极抢抓机遇，主动应对挑战，力创发展顺势。要以办好G20峰会为圆心，以加强供给侧结构性改革、加快“去产能、去库存、去杠杆、降成本、补短板”为重点，坚持实干至上、行动至上，全面推进改革开放和现代化建设各项事业，努力实现“十三五”良好开局。

　　综合各方面因素，建议今年全市经济社会发展主要预期目标为：地区生产总值增长7.5-8%；一般公共预算收入增长8%左右；城镇和农村居民人均可支配收入分别增长7.5%和8.5%左右；居民消费价格涨幅3%左右；节能减排指标完成省下达目标任务。重点做好八项工作：

　　（一）强化峰会服务保障，着力推进城市国际化。办好G20峰会是党中央交给杭州的光荣使命，机遇难得、时间紧迫、责任重大。要以“最高标准、最快速度、最实作风、最佳效果”为要求，全力以赴做好G20峰会服务保障工作，努力呈现历史与现实交汇的独特韵味，真正办成别样精彩、安全圆满的盛会，加快城市国际化进程。高质量完成场馆改造建设和环境整治。确保在6月底前，全面完成峰会主场馆、国宾接待酒店改造提升任务，完成峰会120个环境综合整治重点项目。高标准做好安保维稳和接待服务。深化治安、交通、消防、网络、食品、环境等领域隐患排查和专项治理，依法严打严管严治，有效化解不稳定因素，确保社会安定。做好联络员、翻译员、服务员、驾驶员、志愿者招募培训工作，精心组织礼宾接待等工作。努力提升市民素质和城市文明程度。以“当好东道主、办好G20”为主题，开展市民素质提升行动，弘扬社会主义核心价值观，提升“最美现象”文化品牌，营造全民支持和服务峰会的浓厚氛围，展示“现代、文明、开放、诚信、友善”的杭州市民形象。全面提升城市国际化。抓住峰会契机，对接国际标准，提升城市管理水准。放大峰会综合效应，提升西博会、云栖大会、动漫节、文博会、电商博览会等展会水平，扩大经贸合作与交流。制定实施新一轮旅游国际化行动计划，加大境外推介力度，促进旅游业“四位一体”转型发展。加快卫生等重点领域国际化，创设市一医院、市中医院、滨江医院、下沙医院国际医疗中心。

　　（二）强化改革开放，着力增强体制机制活力。深化政府管理体制改革。深化“四张清单一张网”改革，全面推广市区投资项目并联审批互联互通等制度，推进“先证后照”、“证照网上申请、快递送达”等商事制度改革，健全事中事后监管机制。开展重大财政资金绩效及财政政策评估，推进全过程预算绩效管理。加快实施不动产登记制度改革。建立大数据管理机构。深化经济发展体制改革。推进建设用地、节能权、碳排放权、排污权、水权等资源要素交易，提高资源配置效率。深化国有企业改革，加快资产重组和资产证券化。加大金融改革创新力度，推进钱塘江金融港湾、杭州财富管理中心、全国互联网金融创新中心建设。深化城市管理体制改革。加快推进萧山、余杭、富阳、大江东与主城区一体化融合发展。调整西溪湿地管理体制。出台户籍制度改革整体方案。加快大交通管理体制改革，有序实施传统出租车行业管理体制改革和网络约租车规范管理。扩大对内对外开放。实施跨境电商综试区发展规划，加快“六体系两平台”建设，做大B2B规模。鼓励企业走出去，培育壮大本土跨国企业。办好二十国集团工商界活动（B20峰会），强化招商引资“一把手”工程，创新招商模式，优化招商综合服务，争取更多世界500强、行业领军企业、优质央企、民企和浙商回归项目落地。加强与长三角和杭州都市圈城市合作交流，做好对口帮扶工作，深化区域合作。

　　（三）强化创新转型，着力提升发展质量效益。激发创新动力。以国家自主创新示范区建设为重点，推进城西科创大走廊、城东智造大走廊、钱塘江生态经济带、运河湖滨高端商务带规划建设；深化园区整合提升，加快特色小镇建设，新增市级众创空间20家。实施高新技术企业倍增计划，用好企业研发费用税前加计扣除等政策，推进国家小微企业创业创新基地示范城市建设，新增国家重点扶持高新技术企业150家、科技型初创企业300家。鼓励企业建立研究院。落实“人才新政27条”，汇聚更多高层次人才。增强产业实力。深入实施“一号工程”，更加注重实体经济发展。加强粮食生产功能区和现代农业园区建设，大力发展现代生态循环农业。制定实施万亿级信息经济和六大千亿级产业集群发展配套政策，推进“两化”深度融合，推广工厂物联网和智能制造，深化“四换三名”，实施“机器换人”重点项目500个，培育示范应用企业30家。支持“老字号”“国字号”企业创新发展，提升发展茶叶、丝绸等历史经典产业，加快化纤纺织、轻工等传统优势产业转型升级。深化服务业综合改革国家试点，加快发展现代服务业。推进新型建筑工业化。实施质量标准品牌战略，推进质量强市建设。提升内生能力。实施“去产能”行动方案，推进“僵尸企业”兼并重组和破产清算，淘汰落后产能企业200家以上。落实国家和省“降成本”政策，清理和废除一批市级收费项目，切实减轻实体经济企业负担。鼓励企业通过股改、挂牌、上市、发债等途径扩大直接融资，增强内生发展能力。深化大企业大集团五年行动计划、小微企业三年成长计划，支持“个转企、小升规、规改股、股上市”，加快形成雁阵型企业梯队集群。

　　（四）强化有效供给，着力推进经济平稳增长。提高投资有效性和精准性。加大有效投资力度，确保完成6100亿元投资任务，增长10%以上。注重优化投资结构，启动建设一批大项目，确保产业转型、基础设施、城乡统筹、生态环保、公共服务等重点领域投资保持较快增长。创新政府投融资方式，争取国家和省各类基金、债券和资金支持，放大城市发展基金、产业基金的杠杆效应，大力推进PPP项目。强化要素保障，垦造水田6000亩，完成批而未供土地利用3万亩、城镇低效用地再开发3万亩，加快供而未用土地开发建设。创新产品和服务供给。适应需求变化，加快供给结构调整，扩大有效和中高端供给，提高全要素生产率。大力培育信息、文化、健康、绿色、时尚、品质等消费新热点，开展“消费总动员”活动，全年安排200场以上各类促销活动。推动网络市场健康发展，完善城乡电商流通体系建设，实施“互联网+流通”计划，加快传统商贸转型升级。推动房地产和金融市场健康发展。采取租售并举、加大货币化安置、合理安排经营性土地供应等多种方式，加快“去库存化”，促进房地产市场平稳健康发展。加强企业运行监测预警，化解企业资金链风险，维护地方金融市场稳定。

　　（五）强化统筹协调，着力加快城乡一体化。提升中心城区功能。加大城市总体规划（修订）宣传和执行力度，开展分区规划试点，加强城市设计，强化对城市建筑风貌的规划、管控、修补和保护。加快杭州国际空港改造提升，完成萧山机场公路改建。加快地铁二号线西北段、四号线二期、五号线一期、六号线一期和市域轨道交通临安线、富阳线建设。加强地下综合管廊建设，完成地下空间开发利用350万平方米。深化交通治堵，打通断头路，加快推进快速路和停车场（库）等建设。加强智慧交通建设，优化行车道、人行横道和红绿灯设置，提升支小路通行效率，推进“禁左”措施，强化路面严管严治，加强市民文明交通意识和行为规范教育，提高交通管理科学化水平。加快保障性住房建设和棚户区改造，开工47544套（户），竣工43230套；完成城镇危旧住宅房屋治理改造31.5万平方米；实施“城中村”整治提升，打造“城中村”改造样本。推进智慧城管建设，加强城市综合执法，加大对市容市貌、渣土运输、户外广告、老小区停车、城市养犬、公共场所吸烟等重点领域管理，提高城市管理水平。深化城乡区域统筹发展。实施畅通西部三年行动计划，推进快速路网向绕城外延伸。确保完成富春江船闸扩建等16个项目，加快绕城西复线、千黄高速、临金高速、京杭运河浙江段三级航道整治工程、杭黄铁路等重大项目建设。启动新一轮城乡统筹发展，深化区县（市）协作、联乡结村和结对帮扶。加快四县（市）中心城区和中心镇建设。加强农业农村工作。深化农村产权制度改革，全面推进“三权一房”确权登记颁证工作；探索村级股份经济合作社提升发展机制，壮大农村集体经济。深入实施农民素质提升工程，培养新型职业化农民，加强农业科技创新，提高农业标准化、农机社会化水平，发展农村电商、民宿经济、运动休闲、健康养生等新业态，促进农业增效。加大低收入农户和临近贫困线边缘农户帮扶力度，防止返贫，多途径提升收入水平，促进农民增收。启动新一轮美丽乡村建设，推进农村生态化改造和农村精品线路、精品区块建设；提高农房规划设计水平和建设质量，打造“杭派民居”新农村。

　　（六）强化保护治理，着力加快生态文明建设。坚持“保字为先”。实施环境功能区划管理，建立城市开发边界管理体制，加强淳安省级重点生态功能示范区建设试点，推进沿江沿河沿路生态景观保护和生态廊道建设，确保国家生态市创建通过验收，加快国家生态文明先行示范区建设。坚持“治字当头”。全面攻坚“五水共治”，推进“污水零直排区”全覆盖，确保完成省下达的劣V类水质断面消除任务。加快城镇污水处理厂一级A提标改造和七格四期、之江污水处理厂建设，推进农村农业污染防治，建立农村污水管网维护机制；实施防汛排涝项目200个以上，推进“海绵城市”试点和重点区域建设；实施“强库、固堤、扩排”工程，推进钱塘江干支流堤塘加固提标、苕溪清水入湖河道整治；加快千岛湖配供水等工程建设，完成祥符水厂饮用净水改造一期工程，提升7.2万农村居民饮水安全条件；深化节水型小区（企业）创建。大力实施“五气共治”，萧山余杭富阳三区和四县（市）中心城区建成“无燃煤区”，推进热电锅炉超低排放改造；加强机动车尾气治理，推广机动车使用国Ⅴ柴油；全面完成小燃煤锅炉淘汰或清洁化改造；加强建筑工地全覆盖监管和“餐饮排气”治理。出台“五废共治”行动计划，强化源头减量，深化垃圾“三化四分”工作，推进九峰环境能源等重点项目建设；工业危险废物无害化处置率达到96%，医疗废物集中处置率保持100%；全面推进建筑固废综合利用。坚持“管字从严”。按照环境监管最严格城市标准，以铁的决心、铁的手腕、铁的举措做好环境监管和执法。深化“三改一拆”、“两路两侧”、“四边三化”等专项整治，确保完成“三改”1000万平方米、拆除违法建筑1000万平方米。实施能耗强度、总量和煤炭消费总量“三控”行动，开展热电联产行业综合改造升级行动，推行合同能源管理。坚持“制度为本”。加强生态文明制度建设，完善主要污染物排放收费、排污权交易、生态保护绩效评估考核和责任追究等制度，促进资源节约集约利用。

　　（七）强化民生保障，着力提高共建共享水平。推动更高质量就业。实施“创业就业新政27条”，完成大学生创业三年行动计划，以创业带动就业。推进困难群体就业再就业，及时动态消除城镇零就业家庭。加强社会保障和养老服务。实施全民参保计划，推动参保扩面提质增效，推进萧山、余杭、富阳与主城区社会保险一体化。实施机关事业单位养老保险制度改革。完善城乡“四级救助”网络，关怀智残人士，发展慈善事业，鼓励社会各界参与“春风行动”。加强居家养老服务和农村养老工作，深化公办养老机构改革，支持社会力量兴办养老机构，发展养老产业。扩大高品质教育覆盖面。深入实施“品质教育”七大行动，促进15年基础教育优质均衡发展，推动杭州主城区优质教育资源与萧山余杭富阳三区融合发展，加快向四县（市）延伸。支持社会力量办学，促进民办教育健康发展。加快现代职业教育发展，开展“现代学徒制”国家级试点，深化产学融合、校企合作创新发展，打造“杭州工匠”品牌，推进终身教育。推动市属高校转型发展，加快杭师大二期建设。启动西湖大学、湖畔大学和浙江工程师学院前期规划。提高群众健康水平。联动推进医疗、医药、医保改革，深化医养护一体化服务，完善分级诊疗体系，推进“名院集团化办医”和“双下沉、两提升”全覆盖。加强精神卫生工作，加快杭州老年病医院、精神病医院规划建设。加强计生服务管理，重视妇幼保健，实施一对夫妇可生育两个孩子政策。深化学校体育场地对外开放服务，开展全民健身活动。加强平安杭州建设。加强社区治理和服务创新。创新立体化社会治安防控体系，依法严厉打击违法犯罪活动。加强网络管控服务。抓好公共安全和安全生产管理，坚决防范遏制重特大事故发生。推进国家食品安全城市创建，强化食品药品安全管理。加强法律服务队伍和公共法律服务体系建设，完善人民调解、行政调解、司法调解联动工作体系，做好信访工作，化解社会矛盾，提高人民群众安全感。

　　（八）强化政府自身建设，着力提升行政效能。提高素质本领。积极适应发展新常态，加强各级政府机关工作人员的思想建设和素质能力建设，不断提升理论素养、专业水平、法治意识，努力建设一支政治坚定、能力过硬、作风优良、奋发有为的高素质公务员队伍。提高依法行政水平。推进决策科学化、法制化，规范执法，推进政务公开。加强政府立法，依法履行政府职能，完善依法行政制度。依法接受市人大及其常委会的监督，主动接受市政协的民主监督，自觉接受司法、审计、舆论和社会监督。提高治理能力。加强电子政务建设，整合政务大数据，推进政府服务管理创新。建设统一政务咨询服务和投诉举报平台。加大非涉密部门预决算和财政拨款“三公”经费预决算公开力度。提高政府资产配置效率，打造节约型政府。完善政府购买服务目录，提高公共服务供给效率。深化作风建设。巩固“三严三实”专题教育成效，编制首轮政府绩效管理规划，深化综合考评和“公述民评”活动，落实“容错免责”实施办法，推进作风建设常态化、长效化。坚守政治纪律，严守法纪规矩，确保政令畅通，健全廉政风险防控机制，坚决整治“四风”，坚决整肃庸政懒政怠政，坚决惩处各类腐败行为。

　　深入开展“双拥共建”活动，加强国防教育和国防后备力量建设，促进军民融合发展。支持工会、共青团、妇联等人民团体和社会组织充分发挥作用。继续做好外事侨务、港澳台事务、民族宗教、统计、审计、地方志、红十字、残疾人等工作，推动各项事业发展。

　　各位代表，办好民生实事是我市坚持多年的一项重要制度，按照民生实事群众提、群众定的理念，确定了今年十个方面实事，我们将健全工作机制、全力把民生实事办实办好。一是加大水环境治理力度。治理农村生活污水160个村，全面完成全市农村生活污水治理工作；完成河道清淤综合整治240公里；完成道路和住宅小区积水治理100处；完成截污纳管项目200个。二是加大雾霾治理力度。削减挥发性有机物排放3000吨以上，回收油气800吨以上，新增新能源汽车7500辆；加强建筑工地扬尘治理，创建文明施工示范工地160个以上。三是持续改善交通出行。新辟和优化公交线路45条；增扩容公共自行车服务点80处，更新公共自行车10000辆；新建停车泊位48000个。四是改善城乡人居环境。新增管道燃气用户4万户；完成公厕提升改造119座；新增城市绿地450万平方米；推出实物配租房源4500套；完成农村住房改造13460户、农村困难家庭危房改造1260户。五是完善社会保障体系。城乡最低生活保障标准额提升10%以上，农村低保标准达到城镇标准的90%；持社会保障卡人数达900万以上，社会保险全民参保登记率100%。六是加快电商网络建设。新建电子商务乡村服务站（点）1000个；新建E邮站站点300个。七是强化食品安全治理。中小学校大宗食材统一配送或定点采购率达98%以上，阳光厨房建设率达88%以上；建成150家农贸市场快速检测室和40个基层食品安全快速检测室；新增“五可阳光餐饮”400家。八是加强养老为老服务。组织企退人员文体活动900场以上，为30万名以上企退人员开展健康体检；改造提升50家居家养老服务照料中心和50家养老机构。九是丰富城乡文体生活。建设农村文化礼堂100个，新增社会体育指导员1200名，免费无线网络覆盖1500个社区(村)公共文化场地。十是优化公安办证服务。推出省内户籍人员办理出国境证件“一证通”服务；建立公安行政服务办理移动平台、机动车驾考预约和移动教育平台。

　　各位代表，杭州的未来充满希望，干在实处永无止境、走在前列要谋新篇。让我们更加紧密团结在以习近平同志为核心的党中央周围，在浙江省委、省政府和中共杭州市委的坚强领导下，振奋精神、敢于担当，真抓实干、奋发有为，为率先高水平全面建成小康社会、努力建设世界名城而共同奋斗！
